# How do information strategy and information technology governance influence firm performance?

**DOI:** 10.3389/fpsyg.2022.1023697

**Published:** 2022-12-13

**Authors:** Fanlin Wang, Jianing Lv, Xiaoyang Zhao

**Affiliations:** ^1^School of Accounting, Capital University of Economics and Business, Beijing, China; ^2^School of Business Administration, Huaqiao University, Quanzhou, China

**Keywords:** information strategy, IT governance, firm performance, synergistic effects, IT investment

## Abstract

Organizations today engage in turbulent competition to seize opportunities and cope with challenges by making strategy planning, increasing information technology (IT) investment, and other means. Based on survey data through questionnaires, this paper constructs models to explore the synergistic effects of information strategy (IS) and IT governance (ITG) on firm performance. The results show that, first, ITG and IS as explanatory variables have a significant influence on firm performance. Second, ITG has a positive effect on the relationship between IS and firm performance. This study extends existing research on IS and ITG by exploring the synergistic effects of IS and ITG on firm performance. The conclusion provides management insight and practical guidance for enterprises by actively implementing IS to improve firm performance to transform from the inherent pattern of traditional governance to the new technology governance.

## 1 Introduction

Information technology (IT) performance has always been the focus of information system (IS) research (Ravichandran and Liu, 2011). Companies have invested a lot in IT investment over the past decades. However, IT performance varies greatly from company to company (Mithas et al., 2012; Ilmudeen et al., 2022). Earlier studies have debated the so-called “productivity paradox” concerning the ambiguous impact of IT investments on firm performance (Takeda et al., 2021), but the intricate relationship between IT investment and corporate performance remains to be explored. For less-developed economies, in particular, several recent studies have shown a less positive relationship between IT investment and performance in developing countries (Lee and Kim, 2006). According to the research data of the international data corporation (IDC), many enterprises have increased their investment in IT. Whether the increasing investment in IT projects has a positive or negative effect on the improvement of firms’ performance is academically controversial. Part of the related literature has proven a significantly positive effect of IT investment on firms’ financial performance because of the improvement of scientific decisions by IT systems (Ji et al., 2019; Dong et al., 2021; Alfalah et al., 2022). While other studies on the impact of IT investments show weak or non-existent links between IT investment and firm productivity. For example, Ru et al. (2018) find that the IT investment for vendor-managed inventory is not obvious based on anecdotal evidence and empirical studies. Soh and Markus (1995) state that IT investment cannot be directly transformed into corporate performance, only when IT investment is first transformed into IT assets can IT have an impact on corporate performance. From the perspective of resource input and output, based on different research situations, different conclusions have been obtained. Therefore, relevant empirical evidence needs to be further enriched.

To achieve organizational goals, IT in an organization is not enough if it is only regulated, but IT must be managed professionally. Earlier studies found that many organizations have taken note of the concept of IT governance (ITG) in order to justify IT investments (Mikalef et al., 2021; Ali et al., 2022). Because ITG can balance the interests of all parties and enables IT investments to be effective, the matching of IT goals and enterprise goals is a part of corporate governance (Simonsson et al., 2010). Lunardi et al. (2014) found that ITG adaptation had a positive impact on IT investment performance by measuring the pre-adaptation and post-adaptation performance of Brazilian organizations. Several researchers have done works that are associated with the relationship between ITG and corporate performance. For instance, Prasad et al. (2012) suggest that ITG structures contribute to firm performance through IT-related capabilities that improve the effectiveness and efficiency of internal business processes. Prasad and Green (2015) found evidence of a positive association between these ITG considerations and overall firm performance. In addition, Wu et al. (2015) find that strategic alignment can mediate the effectiveness of ITG on organizational performance. Meanwhile, several practical cases show that firms with stronger ITG are more likely to possess business and IT knowledge to nurture organizational learning.

In addition, ITG can promote IT resources to support enterprise performance according to the established goals and improve the company’s cost structure and revenue level, which will increase with IT inputs’ increasing (Aubert and Rivard, 2020). Based on the resource-based view (RBV), IT investment is explicitly included as the resource that strategic IT alignment as a capability can inherently help leverage (Sabherwal et al., 2019). In other words, the essence of ITG is the mechanism of information strategy (IS) and ITG (Sheppard, 1990). Specifically, IS determines the direction and goal of the enterprise’s informatization, while ITG promotes the effective implementation of IS. Companies invest in IT mainly for strategic and efficiency considerations (Ahmad and Arshad, 2014; Takeda et al., 2021). First, managers need to decide how to allocate spending on IT, such as advertising and research and development investment (Mithas et al., 2012; Witra and Subriadi, 2022). IT investment can produce a sustainable competitive advantage, and along with the governance of IT investment, enterprise performance is improved. Specifically, IS and ITG jointly contribute to the realization of corporate governance goals. Second, performance improvement needs effective resource management and process supervision, otherwise, the input–output effect of IT cannot be guaranteed. The effective use of IT, however, relies heavily on good ITG (Wu et al., 2015). When IT and corporate governance go awry, the goal of the company can be devastating. Therefore, people should consider the consistency of IT resources and enterprise goals as well as the synergy of daily management activities from the perspective of ITG. Given that the desired outcome of effective ITG is to achieve the congruence between information strategies and corporate objectives, however, few research papers have theoretically and empirically examined the effect of ITG and IS on firm performance.

Prior literature (Ahmad and Arshad, 2014; Wu et al., 2015; Ru et al., 2018; Ji et al., 2019; Aubert and Rivard, 2020) generally discusses the economic consequences of IT investment behavior or investment of technical factors, which agree that IT as a resource improves firm performance. Furthermore, the literature on ITG generally shows that ITG plays a positive role in improving firm performance (Wu et al., 2015; Aubert and Rivard, 2020). Only a few studies discuss business and IT strategy alignment (Dairo et al., 2021), the relationship between IT investment and strategic alignment (Sabherwal et al., 2019), and the mediation of strategic alignment in mechanisms that ITG affects organizational performance (Wu et al., 2015). However, the related literature lacks the macro-level thinking of strategy’s impact on corporate governance. Specifically, existing research has not explored the impact of ITG and IS on corporate performance. Moreover, the data from the first-hand data directly obtained from the enterprise to consider the IS and ITG are scarce. In order to develop a richer understanding of the relationship between ITG, IS, and firm performance, this study focuses on how ITG influences firm performance and how the alignment effect of ITG and IS affects firm performance.

This study provides research contributions in several primary ways. First, this study extends prior research on firm strategy and firm performance. Existing research mainly focuses on business and IT strategy alignment (Dairo et al., 2021), the relationship between IT investment and strategic alignment (Sabherwal et al., 2019), discusses the mediation of strategic alignment in mechanisms that ITG affects organizational performance (Wu et al., 2015), and lacks research that explores the impact of IS on firm performance from an independent perspective. This study constructs the measure of IS and tests the impact of IS on firm performance. Furthermore, we explore the influence of IS on enterprise performance from different aspects. This paper focuses on the IS of enterprises, which provides a certain reference value for the application of IS research in organizational performance management. Second, this study adopts the questionnaire data from Chinese enterprises to empirically analyze the impact of informatization strategy and ITG on enterprise performance. Specifically, the data from the first-hand data directly obtained from the enterprises to construct the measure of the IS and ITG are scarce. To fill this research gap, we construct empirical measures for ITG and IS. Third, although most studies related to ITG have explored the relationship between ITG and organizational behavior (Wu et al., 2015; Tawafak et al., 2020), the study still rarely connects IT strategy, ITG, and corporate performance in the same research framework. We also formulate a framework to connect together ITG, IS, and firm performance. Given that ITG and IS have rarely been studied together, their joint relationship in promoting firm performance remains theoretically underdeveloped. The results can provide evidence of the economic consequences of IS under the regulation of ITG, which would inspire Chinese enterprises to adjust current strategies and governance to realize sustainable development.

The remainder of this paper proceeds as follows. The section following reviews the theoretical literature, employing prior literature on ITG, IS, and firm performance, and this section also develops the hypotheses to test the main idea of this study. Subsequently, the research methodology, data collection procedures, variable measurement, and the respondent sample are described. The results of construct validation and model testing are then reported. The paper concludes with a summary of the study findings, highlighting contributions, implications, limitations, and directions for future research.

## 2 Literature review and hypotheses

### 2.1 Information strategy and firm performance

Information for an organization is indispensable, which can be used as input in decision-making in order to solve problems faced by the organization (Astuti, 2018). With the upgrading of IT systems, the company will adjust its strategy, which is a basic change in strategic orientation. For example, an ERP is an IS that brings about radical changes within organizations, changing both the IS environment and overall corporate business process, which may influence the organization’s performance (Park, 2018). Thus, the strategy is crucial in digital time (Proksch et al., 2021). Because corporate strategy is related to IT mechanisms, a good IS can improve corporate performance (Dong et al., 2021). According to the theory of management experts, such as Porter and Mintzberg, IS should be mentioned together with the corporate general strategy. Thus, many scholars have verified their conclusions through case studies or empirical methods. For example, Jeffers et al. (2008) found that IT can contribute to enhancing the value of the firm *via* its strategic role. Mikalef and Pateli (2017) also suggested that IT-enabled dynamic capabilities facilitate two types of agility, market capitalizing and operational adjustment agility, which in sequence enhance firm performance. Dairo et al. (2021) indicate the strategic alignment of IT and business brings many advantages, including enhanced operational efficiencies, business innovativeness, and additional competitive advantage, which together lead to improved performance. In addition, some scholars believe the complementary system of IT resources has significant effects on corporate performance (Cohen and Olsen, 2013). Lin (2022) expands the understanding of IT value by adding a customer-based view (CBV) to the prevalent RBV and indicates that value from IT investments can have direct or indirect effects on firm performance. The overall IS can guarantee the realization of enterprise performance goals in the aspects of organizational design, resource allocation, and management improvement (Cohen, 2008). However, in practice, many enterprises believe that IT behavior is only a matter of the technical department and that only needs to be considered at the functional department level, just like procurement, production, and other activities. According to relevant management practices, this paper believes that IS which is decided by the management level is of great importance. Compared with enterprises without IS, enterprises with IS orientation have better information effects and a better guarantee of enterprise performance by reforming organization structure design, improving resource allocation, and boosting management efficiency. Hence, this paper proposes the following hypothesis:

H1: Information strategy has a positive impact on firm performance.

How to use technology strategically to access and apply information quickly is a serious and profound question when IT is highly pervasive. From the perspective of strategy hierarchical structure, IS includes the company’s general strategy, competitive strategy, and functional strategy (Lederer et al., 1997). Therefore, when formulating IS, enterprises also have three corresponding choices to match up with the three kinds of strategy types. In order to get closer to the actual situation of enterprises and obtain objective research conclusions and further explore how the IS formulation level affects enterprise performance, this paper designs the following sub-hypotheses from three perspectives.

First, IS has a significant effect on competitive advantage (Astuti, 2018). However, ISs could be a source of sustainable competitive advantage only if the IS will be implemented in the enterprises. From the perspective of corporate governance structure, the higher the level of strategy formulation, the more secure the resources to implement the strategy, and the strategy is more likely to succeed. Therefore, if the IS is put forward by the top management of the enterprise, the IS will be in line with the goal of managers, and the strategic goal will be vigorously promoted. It will maximize the important role of information resources and be conducive to the improvement of enterprise performance. Therefore, the first sub-hypothesis is proposed as follows:

H1a: The IS made by the top management will have a positive effect on firm performance.

Second, if a company has a reasonable IS, it must have a thorough and rigorous implementation process, including a comprehensive IT system procurement plan, sufficient talented personnel, technical support, and scientific control of cost, risk, and quality. The control of the process of strategy implementation is as important as the scientific formulation of strategic objectives. As Mohamad et al. (2017) conclude, the enterprise must control the corresponding process in order to improve enterprise performance. According to Nolan’s information implementation model theory (Nolan, 1973), the strategy implementation process is the quantitative change process of informatization, but the strategic goal is the qualitative change process of informatization. Only when the two processes are highly unified, can the information resources be used efficiently, the decision-making quality be upgraded highly and overall performance be improved greatly. Therefore, the second sub-hypothesis is proposed:

H1b: Information strategy with a clear implementation path and reasonable control has a strong effect on firm performance.

Third, according to the theory of corporate governance, the professional background of board members is conducive to understanding and promoting the implementation of strategies (Turedi, 2020; Shreeve et al., 2022). As the IS is more professional and continuous, the implementation is necessary to need managers who have IT professional background. Qualified managers with IT backgrounds can ensure the implementation of IS from a professional perspective and guarantee the maintenance of information results and the use of information resources, so as to improve enterprise performance. Therefore, the third sub-hypothesis proposes the following:

H1c: The IT background of board members has a positive effect on firm performance.

### 2.2 Information technology governance and enterprise performance

The ITG literature provided us with a theoretical basis to investigate how firms effectively build the alignment between IT resources and other resources in creating competitive advantages (Prasad et al., 2012; Wu et al., 2015; Matta et al., 2022). Many companies have spent their resources in order to increase their competitive advantage by controlling their internal processes (Chatzoglou et al., 2011; Wolf and Floyd, 2017). Neff et al. (2013) found ITG is associated with firm performance through IT-relatedness and business process-relatedness. IT-related capabilities also relate to measuring business value at the process and firm levels. This makes it possible to infer that collaborative organizations’ ITG efforts contribute to business value (Prasad et al., 2012). Several studies defined ITG. For example, Benaroch and Chernobai (2017) indicate that “ITG is…an integral part of enterprise governance and consists of the leadership, organizational structures and processes that ensure that the organization’s IT sustains and extends the organization’s strategies and objectives.” The organization defined ITG as the process of controlling IT resources and balancing the interest demands of various stakeholders under the framework of corporate governance (Huang et al., 2010), so as to make the operational goals of information resources consistent with the overall goals of the enterprise (Williams and Karahanna, 2013).

Information technology governance forms an important and integral part of an organization’s corporate governance (Lunardi et al., 2014; De Haes et al., 2019). ITG functions in the same way as enterprise governance to enable an enterprise to more effectively address major business issues such as enterprise resource planning (Lainhart, 2000). Hence, ITG has received increased attention from business practitioners and researchers (De Haes et al., 2019; Matta et al., 2022). Specifically, ITG includes the management mode of technical resources, stakeholder balancing mechanism, technical level, and the alignment of IT and business systems. From the perspective of economics, ITG includes the implementation of IT software, hardware, and other resources, which could lead to the low efficiency and high cost of the company’s short-term business process, and the temporary increase in financial pressure including training costs and error correction cost, but theoretical deduction and practical observation can find that ITG can ultimately improve the overall performance of enterprises (Raymond et al., 2019; Matta et al., 2022).

Information technology governance can improve the company’s performance in two ways. On the other hand, ITG cost, which contains the hardware and software investment, security investment, and technology upgrading investment, belongs to environmental investment. This kind of investment can ensure the normal operation of the enterprise’s IT system, which indirectly improves the company’s performance and directly consumes enterprise resources. At the same time, the integrated IS’s overall function, network bearing capacity, the capability of output loading, and other technical resources play an important role in the enterprise’s process execution and department collaboration. Therefore, ITG will undoubtedly improve the company’s performance. On the other hand, clear and reasonable IT planning is the embodiment of ITG in system service capability. Board-level ITG can improve organizational performance (Matta et al., 2022). More importantly, enterprises can make full use of big data, cloud computing, data mining, and other means to allocate information resources and serve for the improvement of enterprise performance. When an enterprise makes a large IT investment, it is necessary to set up a management team and attach importance to the training of IT skills of employees. Therefore, this process can also improve the professional level and treatment of employees, which is conducive to enhancing the competitiveness of enterprises. In addition, during the construction and operation of the IT system, the business department and IT department need to keep close cooperation. The goal is to embed business or management requirements into IT systems, so as to avoid inadequate implementation in which business and technology are disconnected from each other. Only in this way, the efficiency of business processing, management decision-making, and risk control can be improved, in this process, enterprise performance will also be improved. To sum up, this paper proposes the following hypothesis:

H2: The implementation of ITG has a positive effect on firm performance.

### 2.3 The synergistic effects of information technology governance

Through the analysis of IS and ITG, it can be found that no matter the ITG mechanism in the context of IS (Schlosser et al., 2015), or the formulation of reasonable IS after analyzing the characteristics of ITG, ITG and IS are always the relationship between strategy and tactics. On the one hand, IS provides direction and targets for IT resource governance. Clear goal setting will make ITG more efficient. On the other hand, in order to maintain competitive edges, organizations try to adapt rapidly to digitalization through the transformation of operations and processes (Ho et al., 2022). ITG basically places an outline around how a firm’s IT plan supports a firm plan. This IT-firm configuration will make sure that the business maintains to accomplish its plans and objectives and apply techniques to estimate its performance (Al Romaihi and Hamdan, 2021). Therefore, the depth and breadth of ITG provide implementation support for IS. Strategic alignment and planning have been a top managerial concern since the beginning of the IS profession (Taylor et al., 2010), and prior studies show that ITG mechanisms on organizational performance are fully mediated by strategic alignment (Wu et al., 2015). Therefore, the implementation of an IS can more effectively affect enterprise performance under the influence of ITG. In other words, ITG promotes the promotion of IS to corporate performance, and ITG has synergistic effects on IS’s performance improving effect. Therefore, the following hypothesis is proposed:

H3: Information technology governance has a positive moderating effect on the relationship between IS and firm performance.

## 3 Research methodology

### 3.1 Data collection

Aiming at the influence of IS formulation level and ITG level on enterprise performance, this paper adopted a large-sample questionnaire survey. In the process of questionnaire collection, we made efforts in the following aspects. First, in terms of the subject of investigation, we rely on the China Association of Chief Financial Officers, which is a cross-regional, cross-departmental, and cross-industry national non-profit organization in China to gain the access to distribute questionnaires to 59 group companies and their subsidiaries affiliated to State-Owned Assets Supervision and Administration Commission of China (SASAC). A total of 405 questionnaires were distributed, and 317 valid questionnaires were received during 2016–2018. In 2019, we made a return visit to the relevant companies and added relevant data. The paper questionnaires were distributed to the targeted firms, and then, we collected them later. These firms in Beijing, Shanghai, Guangzhou, Chengdu, Shandong, and other places, the research objects involving managers or directs in manufacturing, Internet, B2B logistics, pharmaceuticals industry, utility industry, and other industries. Second, before distributing questionnaires, we first limited the scope of distributing questionnaires. Specifically, in order to measure the level of ITG and get more information about IS, we choose the firms that have ITG expression in relevant company governance documents. Furthermore, we try to ensure the authenticity and representativeness of the data source; the subjects of the questionnaire are limited to enterprise informatization managers including the CEO, managers of the technical department, or directors of the information center; when a company does not have a technology department, we send questionnaires to managers who are in charge of IT, and we also send questionnaires to financial managers who evaluate enterprise performance. We distributed at least three questionnaires in one firm (the firm refers to group companies or subsidiaries). At the same time, we recheck some financial indexes *via* the China Accounting Informatization Committee and other professional institutions. We also manually checked the relevant financial data with the company’s financial statement. Third, each questionnaire inquired about the financial performance data and the IT investment budget data of enterprises in the past 5 years, and the IT investment budget data are based on the average of the 5 years which is due to the firms usually planning their investments over a 5-year horizon. Finally, the data processing is performed on SPSS 19.0.

### 3.2 Variables

#### 3.2.1 Information strategy

Information strategy stipulates the direction, target, and resource allocation principle of the information scheme (Astuti, 2018). In terms of the decision-making level of the company, IT investment and IS operations are planned and arranged from a higher level. Therefore, the connotation of an IS is abundant and the measurement of an IS is more flexible. In order to accurately grasp the different attributes of IS, this study designs the indicators from the IS formulation level, IS process supervision, and IS result maintenance, specifically including whether the strategy is formulated or approved by the senior leaders of the company (SI1), whether the process supervision of IS touches each important process (SI2), and whether the operation and maintenance of information results are good or not (SI3). All items are expressed on a 10-point Likert scale (where “1” means totally disagree and “10” means totally agree). The specific descriptions are provided in Table 1.

**TABLE 1:** Information strategy evaluation index.

Overall index	Specific index	Index code
Information	Formulated and approved by the board of directors	SI11
strategy	Integrate the needs of all departments	SI12
formulation	Information strategy implement 3 years above	SI13
level (SI1)	Detailed planning and implementation of the program	SI14
	Strategic resource planning at company level	SI15
Information	Reasonable IT system procurement plan	SI21
strategy process supervision (SI2)	Adequate human, technical and management resources	SI22
	Reasonable outsourcing or delegation plan	SI23
	Reasonable implementation process plan	SI24
	Reasonably control the change of management	SI25
	Effectively control the process and results	SI26
Information	Follow-up technical support is incompetence	SI31
strategy results	Independent evaluation of the operation effect	SI32
maintenance (SI3)	Operation risk and safety risk is controllable	SI33

#### 3.2.2 Information technology governance

The definition of ITG from the IT Governance Institute (ITGI) indicates that ITG is an integral part of enterprise governance and consists of leadership, organizational structures, and processes. Moreover, Benaroch and Chernobai (2017) show that ITG consists of the leadership and organizational structures and processes that ensure that the organization’s IT sustains and extends the organization’s strategies. Furthermore, ITG is seen as the organizational capacity exercised by the board, executive management, and IT management to control the formulation and implementation of IT strategy and in this way ensure the fusion of business and IT (Neff et al., 2013). For constructing the measurement of ITG, this study refers to the prior literature. We will evaluate ITG from five dimensions: IT investment, IT technology resource, IT human resource (IHR), IT relationship resource, and IT capability. The index of IT investment (IV) and the sub-item index of IHR are the real value of the subjective firm, and other indexes are expressed on a 10-point Likert scale (where “1” means the level is lowest and “10” means the level is highest). The total indicator (ITG) is taken as a simple average of the sub-indicators (respectively, IV, IR, IHR, IRR, and IC). The specific descriptions are shown in Table 2.

**TABLE 2:** Information technology (IT) governance evaluation index.

Process index	Specific index	Index code
IT investment (IV)	Information technology investment quantity	IV1
	The total investment proportion of IT budget	IV2
	The ratio of IT assets to total assets	IV3
IT technology	Technical performance of independent system	IR1
resource (IR)	Overall function of integrated system	IR2
	Network bearing capacity and output load	IR3
IT human resource	The proportion of IT staff in the total staff	IHR1
(IHR)	The salary level of IT staff	IHR2
	The technical level of IT staff	IHR3
	The business level of IT staff	IHR4
IT relationship	Relationship with business department	IRR1
resource (IRR)	Relationship with partnerships	IRR2
	Degree of information sharing	IRR3
IT capability (IC)	IT planning ability	IC1
	Information utilization ability	IC2
	System maintenance ability	IC3

For the evaluation of IT investment management, the secondary indexes are designed as “IT investment quantity” to reflect the total investment directly used to construct IT during the investigation period. “The total investment proportion of IT budget” reflects the investment intensity of enterprises in IT projects. “The ratio of IT assets to total assets” reflects whether an enterprise is a general enterprise or an IT enterprise. For the evaluation of IT technology resources, this paper chooses the performance and load degree of ISs, including “technical performance of independent system,” “overall function of integrated system,” and “network bearing capacity and output load.” The above indicators can comprehensively reflect the capability provided by the enterprise’s technical resources. For the IT talent indicators, this paper makes a comprehensive survey from the aspect of IHRs, including “the proportion of IT staff in the total staff,” “the salary level of IT staff,” “the technical level of IT staff,” and “the business level of IT staff.” It basically covers the structure of the IT department and examines the comprehensive business and financial capabilities. The evaluation of “IT relationship resource” can examine the ability of various departments to cooperate and share resources in the process of ITG, and its level can reflect the environment and foundation of enterprise ITG. Finally, the measure of IT capacity is measured from three comprehensive indicators, namely “IT planning ability,” “information utilization ability,” and “system maintenance ability.” The IT planning capability represents the planning capability and coverage of the technical framework for the strategy. The operational capability of IT is the implementation carrier of IT planning, and enterprises should have operational organization, job responsibilities, and the capability of system maintenance. IT maintenance ability is the safeguard of administrative effect. The maintenance capability is vital to daily practice, thus, it should also be taken as the evaluation index.

#### 3.2.3 Firm performance

Since the mid-1990s, the SASAC has emerged as a key institution governing firm ownership in China (Wang et al., 2012), SASAC started to use enterprise value-added (EVA) assessment as a performance measure on central enterprises in 2010, to guide the way how enterprises develop. Considering that the subject of the questionnaire is not all listed companies, non-listed companies are also included in the sample, and these firms are affiliated with the SASAC. Therefore, referring to the SASAC’s firm performance evaluation method and according to Du et al. (2012), we construct evaluation indexes from the three dimensions of financial ability, development ability, and social responsibility. The specific definition is shown in Table 3.

**TABLE 3:** Enterprise performance evaluation index.

Evaluation dimensions	Evaluation index	Weight (%)	Calculation formula
Financial capacity	Return on total assets	20	(Net profit + income tax + financial expenses)/Total annual assets
	Total asset turnover	10	Operating revenue/Total annual assets
Development capacity	Earnings multiples	10	(Net profit + income tax + financial expenses)/Financial expenses
	Technology investment proportion	20	Total technical expenses/Operating revenue
	Growth rate of operating revenue	20	Revenue growth of current year/Revenue of last year
Social responsibility	Social expenditure ratio	20	Social expenditure/Operating revenue

We weighted firm performance from three dimensions, financial ability indicators include return on total assets and total asset turnover, with a total weight of 30%. The development capability indexes are used to evaluate the growth of an enterprise, including the solvency guarantee ability, sustainability ability, and the company scale expansion, accounting for 50% of the weight. Social responsibility measures a company’s responsibility for environmental protection, public welfare, and other aspects of social expenditure. This index accounts for 20% of the weight.

### 3.3 Model design

In order to explore the association among ITG, IS, and firm performance using the regression model, according to Hypothesis H1’s each sub-hypothesis and Hypothesis H2, the empirical model was constructed as follows:


Model (1) $$G\,P=\beta_{0}+\beta_{1}\,S\,I\,1+\beta_{2}\,S\,I\,2+\beta_{3}\,S\,I\,3+\beta_{4}\,D\,O\,A+\beta_{5}\,S\,I\,Z\,E$$

$$+\beta_{6}\,△\,G\,P+Y\,E\,A\,R+I\,N\,D\,U\,S\,T\,R\,Y+\varepsilon$$

Model (2) $$G\,P=\beta_{0}+\beta_{1}\,I\,T\,G+\beta_{2}\,D\,O\,A+\beta_{3}\,S\,I\,Z\,E+\beta_{4}\,△\,G\,P+Y\,E\,A\,R$$

+I⁢N⁢D⁢U⁢S⁢T⁢R⁢Y+ε

In the above equation, GP represents the dependent variable firm performance, ITG represents the level of ITG, and SI was represented by the sub-item indexes in order to further explore the different aspects of IS. SI1 represents the IS formulation level, SI2 represents the intensity of IS process supervision, and SI3 represents the capability of IS result maintenance. According to Wu et al. (2015), firm size is usually treated as one of the antecedents to organizational performance, and we model it as a control variable directly affecting firm performance as our main focus. Size is the scale of the enterprise, measured by the logarithm of the number of employees (Wu et al., 2015). We include DOA, which is measured as the leverage of firms as total liabilities to total asset ratio (Mahmood et al., 2019). We also consider the potential influence that IT investment and enterprise performance are mutually influenced, as firms with better performance in the past may have more resources to devote to IT function (Turedi, 2020), thus, we controlled ΔGP. We also include the year (Year) which is a dummy variable to control the implicit influence that can change over time. Xue et al. (2012) also found that the impact of IT asset portfolios on organizational efficiency varies in different industrial environments. We control industries classified by the China Securities Regulatory Commission industry in 2012. Furthermore, due to the limited variable measures that can be obtained from the questionnaire, we do some efforts when selecting the sample of the questionnaire. For example, as mentioned above, the respondents of the questionnaire survey are the companies that mention ITG in their corporate governance documents, in order to control more corporate characteristics that affect firm performance. β_0_ is a constant term. ε is the error term. In Model (1), if β_2_, β_3_, and β_4_ are significant, Hypothesis H1 and the sub-hypotheses are supported. β_1_ represents the impact of ITG on corporate performance. In Model (2), if β_1_ is significant, Hypothesis H2 is supported.

To test the Hypothesis H3 and to explore whether ITG has a positive moderating effect on the relationship between IS and firm performance, the empirical model was constructed as follows:


Model (3) $$G\,P=\beta_{0}+\beta_{1}\,I\,T\,G+\beta_{2}\,S\,I\,1+\beta_{3}\,S\,I\,2+\beta_{4}\,S\,I\,3+\beta_{5}\,S\,I\,4$$

$$+\beta_{6}\,I\,T\,G\times S\,I\,1+\beta_{7}\,I\,T\,G\times S\,I\,2+\beta_{8}\,I\,T\,G\times S\,I\,3$$

$$+\beta_{9}\,D\,O\,A+\beta_{10}\,S\,I\,Z\,E+\beta_{11}\,△\,G\,P+Y\,E\,A\,R$$

+I⁢N⁢D⁢U⁢S⁢T⁢R⁢Y+ε

In Model (3), the control variables are as same as in Model (1). ITG × IS1, ITG × IS2, and ITG × IS3 are interactive terms. If β_2–_β_5_ are significant, coefficients of estimation are positive, and if β_6–_β_8_ are significant, coefficients are also positive; it indicates that ITG has a positive moderating effect on the relationship between IS and firm performance. Hence, Hypothesis H3 is supported.

### 3.4 Index reliability and validity analysis

In order to measure the reliability of the evaluation system, the method is to calculate the Cronbach reliability coefficient. The formula is as follows:


(1) $$C\,r\,o\,\alpha =\frac{K}{K-1}\,\left(1-\frac{\sum S_{i}^{2}}{S^{2}}\right)$$

where *K* represents the number of scale questions; $\sum S_{i}^{2}$is the sum of the variance of items in the scale; *S*^2^is the variance of the items added together.

According to the estimation experience of previous scholars, the closer the Cronbach coefficient is to 1, the higher the representativeness and stability of the scale and the more reliable the evaluation value will be. When the coefficient exceeds 0.9, the scale is considered to have high internal reliability. If it is between 0.8 and 0.9, it could be accepted in a higher range. If it is between 0.7 and 0.8, the scale design is defective. However, it also has a certain reference value. If the Cronbach coefficient is less than 0.7, the scale design is unsuccessful and needs to be redesigned. The formula shows that the Cronbach coefficient will increase with the increase in *K*-value. In order to achieve objective results, more indicators are designed for the same project. According to the above-mentioned formula, the Cronbach coefficient can be calculated to be 0.839, which is in an acceptable high range, so the scale system is reliable and stable.

We also do some effort to verify the validity of this questionnaire. First, in the process of developing this questionnaire, the professionals in the China Association of Chief Financial Officers from different provinces help us to assess the items of the questionnaire. We have revised the statement of the corresponding question according to the opinions of experts. Second, we tested the face validity of the test. The enterprises taking the questionnaire test were asked to participate in a study at the beginning of the test to complete a short questionnaire regarding the face validity of the test. The test results show that the questionnaire is valid.

**FIGURE 1:**
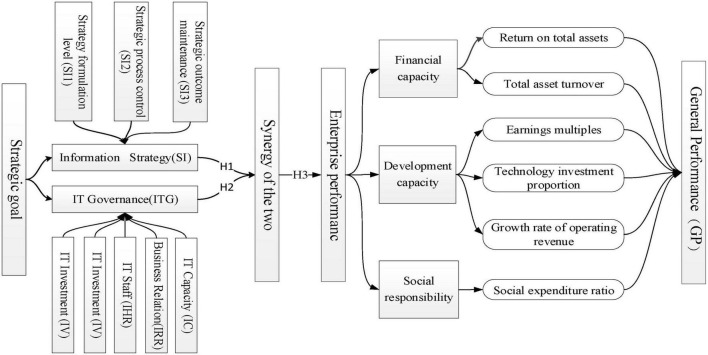
A research framework.

In order to further investigate how IS and ITG affect firm performance, we follow the research framework as is shown in the elements of Figure 1. As shown in Figure 1, IS is divided into three categories according to the implementation process, which are represented by SI1, SI2, and SI3, respectively. ITG is decomposed into five categories, represented by IV, IR, IC, IHR, and IRR, respectively. At the same time, the relations are shown. For example, H1 represents the impact of IS on enterprise performance. H2 represents the impact of ITG on enterprise performance. H3 represents the synergy impact of ITG and IS on enterprise performance.

## 4 Empirical analysis

### 4.1 Descriptive statistics

The mean, minimum value, maximum value, and standard deviation of each study variable are shown in Table 4. The mean return on total assets in the sample is 3.21%, which is close to the statistics of Chinese firms’ return on total assets in the previous study (Giannetti et al., 2021). SI has a mean of 3.68, which indicates that the sample firms’ implementation of the IS level is not mature enough. ITG has a mean of 4.06, and the standard deviation is 0.31, which means that the difference in ITG level among sample firms is less.

**TABLE 4:** Descriptive statistics.

	Min	Max	Mean	SD
General performance	0.37	4.91	1.88	1.17
Return on total assets	0.16%	11.90%	3.21%	0.03
Total asset turnover	0.05	0.86	0.2816	0.29
Earnings multiples	1.49	30.79	8.7000	7.77
Technology investment proportion	4.62%	909.39%	142.58%	2.20
Growth rate of operating revenue	21.78%	87.79%	58.23%	0.20
Social expenditure ratio	0.01%	4.07%	0.68%	0.01
IT governance (ITG)	3.54	4.63	4.06	0.31
IT investment (IV)	0.08	0.30	0.18	0.07
IT technology resource (IR)	3.17	4.32	3.81	0.24
IT human resource (IHR)	3.01	4.82	3.86	0.63
IT relationship resource (IRR)	3.86	4.74	4.19	0.27
IT capability (IC)	3.33	4.67	3.92	0.34
Effect of information strategy (SI)	2.69	5.07	3.68	0.73
Information strategy formulation level (SI1)	2.47	4.60	3.32	0.63
Information strategy process supervision (SI2)	2.33	4.33	3.18	0.63
Information strategy results maintenance (SI3)	3.27	6.27	4.55	0.92

### 4.2 Correlations

First, we conduct a correlation analysis on the hypothesis and the correlation results are shown in Table 5. There is a significant positive correlation between IS formulation level (SI1) and firm performance (*r* = 0.807, *p* < 0.01), between IS process supervision (SI2) and firm performance (*r* = 0.850, *p* < 0.01), and between IS result maintenance (SI3) and firm performance (*r* = 0.836, *p* < 0.01). This finding suggests that IS has a positive impact on firm performance. The IS made by the top management has a positive effect on firm performance and the IS which has a clear implementation path and reasonable control has a strong effect on firm performance. The IT background of board members also has a positive effect on firm performance. In other words, the three sub-hypotheses of H1a–H1b are preliminarily supported.

**TABLE 5:** Information strategy and corporation performance.

Index		Strategy level (SI1)	Process supervision (SI2)	Results maintenance (SI3)
General	Pearson correlation	0.807**	0.850**	0.836**
performance (GP)	Significance (bilateral)	0.001	0.002	0.000
	*N*	317	317	317

**Correlation is significant at the 0.01 level (two-tailed).

Second, this paper mainly focuses on the indicators of ITG which cover a firm’s IT investment, IT technology resources, IHRs, IT relationship resources, and IT execution ability. The correlation results between ITG and firm performance are shown in Table 6. There is a significant positive correlation between information governance (ITG) and firm performance (*r* = 0.911, *p* < 0.01). Therefore, Hypothesis H2 is supported. It shows that technology, talent, business integration, and technical ability can promote enterprise performance.

**TABLE 6:** Information strategy, IT governance, and corporation performance.

		General performance (GP)	Information strategy (SI)	IT governance (ITG)	SI × ITG
GP	Pearson correlation	1	0.910**	0.911**	0.9430**
	Significance (2-tailed)	0.000	0.000	0.000	0.000
	*N*	317	317	317	317
SI	Pearson correlation	0.910**	1	0.961**	0.999**
	Significance (2-tailed)	0.000	0.000	0.000	0.000
	*N*	317	317	317	317
ITG	Pearson correlation	0.911**	0.961**	1	0.999**
	Significance (2-tailed)	0.001	0.000	0.000	0.000
	*N*	317	317	317	317
SI × ITG	Pearson correlation	0.943**	0.999**	0.999**	1
	Significance (2-tailed)	0.000	0.001	0.000	0.000
	N	317	317	317	317

**Correlation is significant at the 0.01 level (two-tailed).

Third, we also conduct a correlation analysis to analyze the moderating role of ITG. We conduct a correlation analysis on IS, ITG, and corporation performance. The results are shown in Table 6. We mainly focus on the moderating role of ITG on the impact of IS on firm performance; the cross-multiplication term of SI and ITG is used to express the impact of the joint action of IS and ITG on enterprise performance. There is a significant positive correlation between the interaction term (SI × ITG) and firm performance (*r* = 0.943, *p* < 0.01), and this correlation is significant at the 1% level, with a positive correlation and a correlation coefficient above 0.9. Therefore, this result shows that ITG can strengthen the positive relationship between ITG and firm performance. Hypothesis H3 is preliminarily supported.

### 4.3 Regression analysis

#### 4.3.1 Information strategy and firm performance

Through the correlation analysis of enterprise performance (GP), SI, and ITG in the sample, the correlation coefficient is found to be greater than 0.8. Although it can be explained that they have a correlation in statistical attributes, it is impossible to deeply understand the causal relationship behind them and the specific performance of various factors. Therefore, the ordinary least square linear (OLS) regression model is used in this paper to reveal the causal relationship and logical cause between variables.

We conduct an empirical analysis on Hypotheses H1a–H1b and the regression results are shown in Table 7. Model (1) shows that the coefficients of SI1, SI2, and SI3 are 0.357, 0.430, and 0.625 and are significant at the 1% level or 5% level. This finding suggests that the IS made by the top management, an IS that has a clear implementation path and IT background of board members, has a positive effect on firm performance, which means that the information strategy has a positive effect on firm performance. Therefore, Hypothesis H1 and three sub-hypotheses are supported.

**TABLE 7:** Information strategy and firm performance.

Variables	Dependent variable: Firm performance
	Model 1
C	0.961*** (0.017)
IS1	0.357*** (0.131)
IS2	0.430** (0.195)
IS3	0.625** (0.303)
DOA	−0.104** (0.052)
Size	−0.310** (0.132)
ΔGP	−0.042* (0.024)
Year	Yes
Industry	Yes
*R* ^2^	0.208
*N*	317
*F*	18.547***

The data are the SPSS processing output of the questionnaire data. ^***^Significant at the level of 0.01. ^**^Significant at the level of 0.05. *Significant at the level of 0.10. The numbers in parentheses are standard errors.

#### 4.3.2 Information technology governance and firm performance

We conduct regression analysis according to Model (2) to test the relationship between ITG and IT performance, and the results are shown in Table 7. In the first column of the regression results, the result shows that ITG has a significant positive impact on firm performance, and the coefficient of ITG is 0.723 and significant at the 5% level. This result shows that ITG contributes to firm performance positively. Our empirical regression results support Hypothesis 2.

#### 4.3.3 The synergistic effects of information technology governance

A regulatory regression Model (3) is constructed to further test the regulatory effect of ITG on the relationship between IS and corporation performance. We add the interactive term of explanatory variable and moderator variable in the regression. By testing the coefficient of the interaction term, we can test the moderating role of ITG. The results are shown in Table 8. The coefficients of the interaction items between ITG and IS formulation level (SI1) and information result maintenance capability (SI3) are 1.032, 0.109, and 1.625 and are significant at the 1, 10, and 5% levels. The coefficient of the ITG is 0.436 and significant at the 5% level. It shows ITG has a moderating effect on IS. The higher the strategic level and the more attention to the maintenance of strategic results and the later assessment, the more conducive to improving enterprise performance. Hypothesis 3 is supported.

**TABLE 8:** Information strategy, IT governance, and firm performance.

Variables	Dependent variable: Firm performance	
	Model 2	Model 3
C	0.626*** (0.170)	0.725*** (0.216)
ITG	0.723** (0.313)	0.436** (0.220)
IS1		0.329** (0.149)
IS2		0.235* (0.139)
IS3		0.316** (0.158)
ITG × IS1		1.032*** (0.357)
ITG × IS2		0.109* (0.063)
ITG × IS3		1.625** (0.674)
DOA	−0.098** (0.045)	−0.102** (0.051)
Size	−0.234* (0.134)	−0.36** (0.181)
ΔGP	−0.019** (0.009)	−0.021** (0.009)
Year	Yes	Yes
Industry	Yes	Yes
*R* ^2^	0.226	0.224
*N*	317	317
*F*	19.225***	16.013**

The data are the SPSS processing output of the questionnaire data. ^***^Significant at the level of 0.01. ^**^Significant at the level of 0.05. *Significant at the level of 0.10. The numbers in parentheses are standard errors.

#### 4.3.4 Discuss the endogenous

The relationship between the implementation level of IS and firm performance or the relationship between ITG and firm performance may not be caused by the impact of IS or ITG, but the other important omitted variables or they have a mutual cause-and-effect relationship. Specifically, we take some efforts to alleviate omitted variables problems. First, in terms of avoiding omitted variable bias, some considerations have been made in the early stage of the study. On the one hand, we randomly selected the interviewee from the alternative companies which are restricted to having certain characteristics. According to the above description of the selection process of respondents for the questionnaire, we randomly select the firm in which corporate governance documents have some ITG-related statements. The inclusion of ITG in corporate governance documents indicates that these companies have a deeper understanding of the concepts of IT investment, ITG, informatization, and so on. Therefore, these companies have more similar characteristics in IT. We randomly selected subjects from these representative firms and handed out questionnaires anonymously. We try to use this method to mitigate the problem of important omitted variables. On the other hand, in the stage of questionnaire design, we seek the opinions of authoritative experts to ensure the comprehensiveness of the index system, and then we can avoid the negative impact of omitted variables in the model design. Second, we conducted interviews with enterprise professionals to confirm whether there is a mutual cause-and-effect relationship between investment in IT (including IT investment, the formulation of informatization strategy, and setting up a corresponding IT department in the organization) and firm performance. In fact, in the first survey, we focused on the problem. Those surveyed believe that if IT investment does not contribute to enterprise performance, the companies will not continue to invest for 3 or even 6 years in IT even if they have more strength. Therefore, the empirical results are credible. It shows that IT investment and enterprise performance are not cause-and-effect relationships.

## 5 Discussion

### 5.1 Conclusion

Through the above theoretical analysis and empirical discussion, the following conclusions and enlightenment are obtained. First, when the total amount of information resource input of an organization remains unchanged, different levels of IS have different impacts on firm performance. The higher the level of strategy formulation, the greater the impact on enterprise performance. The principle of “top management” is more suitable for the information work of modern enterprises. Second, the higher the level of ITG, the more conducive to the implementation of IS excluding the control factors such as enterprise scale and organizational structure. The IS approved by the company’s top management ensures the authority of investment from the organizational level. However, it also needs to be bound with the process participation of the units and functional departments. The synergy effect between IS and ITG has been well verified in the above empirical processes. Third, in view of the important role of ITG in ensuring the implementation of IS, it is suggested that some board members of IT enterprises should have corresponding IT backgrounds, and the companies should add ITG departments and posts to ensure the effective management of core IT resources.

### 5.2 Theoretical contributions

Information technology investment, ITG behavior, and IS in the era of the digital economy have become a hot research topics. Existing research mainly discusses the impact of IT investment and ITG on firm performance. However, the impact of IS on firms’ performance is still rare, and how ITG and IS influence firm performance has not caught attention. Therefore, the theoretical contributions of this study are mainly in two aspects: First, existing research mainly focuses on the relationship between IT investment and strategic alignment (Sabherwal et al., 2019) or discusses the mediation of strategic alignment in mechanisms that ITG affects organizational performance (Wu et al., 2015), prior literature not discussed separately from the aspect of IS perspective. This study constructs the measure of IS and tests the impact of IS on firm performance which extends prior research on firm information strategies. Furthermore, we further explore the influence of IS on enterprise performance from different aspects. This paper focuses on the IS of enterprises, which provides a certain reference value for the application of IS research in organizational performance management. Second, this paper emphasizes the interaction effect of ITG and IS on firm performance from the perspective of strategy formulation and control efficiency instead of considering the impact of the two elements on corporate performance separately as most previous studies do. Although most studies related to ITG have explored the relationship between ITG and organizational behavior (Wu et al., 2015; Tawafak et al., 2020), the study still rarely discusses the moderating role of ITG, and we find that ITG can strengthen the positive relationship between IS and firm performance by monitoring strategy formulation and improving control efficiency. This paper provides empirical evidence for corporate governance from the perspective of ITG. Third, this study adopts the questionnaire data from Chinese enterprises to empirically construct the measure of the IS and ITG. This provides a reference for further discussion of the economic consequences of IS and ITG.

### 5.3 Practical contributions

This study provides new insights and solutions for promoting the firm performance. Strategy is the target setting for an enterprise to develop and maintain its competitive advantage. As an important factor in the enterprise’s development, the impact of ITG and IS can play a vital role in improving firm performance. First, with the development of the digital economy and dramatic changes in organizational structure, IT investment performance becoming more and more important, as a strategic goal and means to achieve IT performance, the formulation of IS and ITG is vital for firms to realize high quality development. Second, this paper shows that the IT background of board members has a positive effect on firm performance. Organizations should adjust the structure of management teams according to the development of the IT environment, so as to play a beneficial role in improving enterprise performance. The strategy formulated by the senior management of the organization can ensure the effective implementation of the strategy that inspires the formulation of an IS that is more suitable for the way from “top to bottom” in the strategic decision hierarchy. Third, this paper shows that the higher the level of ITG, the more benefits to IS’s implementation effect. ITG is helpful for the company to realize the goal of IS. Therefore, the importance of ITG should be fully realized when improving the level of corporate governance. Overall, the realization of IS needs the coordination of technology and management wisdom, and at the same time, the idea of system planning should be infiltrated into organizational behavior in order to help organizations achieve the state of modern information management.

### 5.4 Limitations and future research

This study also has some limitations. First, since ITG and IS are difficult to measure, data can only be obtained through questionnaires. In the future, the ITG index and IS implementation index should be further constructed to conduct a more in-depth large-sample test on this issue. Second, this paper only examines the moderating effects of IS and ITG on firm performance but has not yet examined the mechanism of how IS affects firm performance. Future research should explore this issue, so as to open the black box of how corporate strategy setting and corporate governance affect firm performance and firm’s sustainable development in the digital time, so as to provide a useful reference for enterprises to improve IT performance and financial performance.

## Data availability statement

The raw data supporting the conclusions of this article will be made available by the authors, without undue reservation.

## Ethics statement

Ethical review and approval was not required for the study on human participants in accordance with the local legislation and institutional requirements. Written informed consent from the patients/participants or patients/participants legal guardian/next of kin was not required to participate in this study in accordance with the national legislation and the institutional requirements.

## Author contributions

FW and JL conceived and designed the experiments, collected and interpreted the data. XZ analyzed the data, examined and critically contributed to the study, and finally approved the manuscript. All authors contributed to the article and approved the submitted version.
